# Hesperidin Inhibits Inflammatory Response Induced by *Aeromonas hydrophila* Infection and Alters CD4^**+**^/CD8^**+**^ T Cell Ratio

**DOI:** 10.1155/2014/393217

**Published:** 2014-05-06

**Authors:** Abdelaziz S. A. Abuelsaad, Gamal Allam, Adnan A. A. Al-Solumani

**Affiliations:** ^1^Department of Microbiology (Immunology Section), College of Medicine, Taif University, Taif 21974, Saudi Arabia; ^2^Department of Zoology, Faculty of Science, Beni-Suef University, Beni-Suef, Egypt; ^3^Department of Pediatric, College of Medicine, Taif University, Taif 21974, Saudi Arabia

## Abstract

*Background*. *Aeromonas hydrophila* is an opportunistic bacterial pathogen that is associated with a number of human diseases. Hesperidin (HES) has been reported to exert antioxidant and anti-inflammatory activities. *Objectives*. The aim of this study was to investigate the potential effect of HES treatment on inflammatory response induced by *A. hydrophila* infection in murine. *Methods*. *A. hydrophila*-infected mice were treated with HES at 250 mg/kg b.wt./week for 4 consecutive weeks. Phagocytosis, reactive oxygen species production, CD4^+^/CD8^+^ T cell ratio, and CD14 expression on intestinal infiltrating monocytes were evaluated. The expression of E-selectin and intercellular adhesion molecule 1 on stimulated HUVECs and RAW macrophage was evaluated. *Results*. Percentage of CD4^+^ T cells in the intestinal tissues of infected treated mice was highly significantly increased; however, phagocytic index, ROS production, CD8^+^ T cells percentage, and CD14 expression on monocytes were significantly reduced. On the other hand, HES significantly inhibited A-LPS- and A-ECP-induced E-selectin and ICAM-1 expression on HUVECs and ICAM-1 expression on RAW macrophage. *Conclusion*. Present data indicated that HES has a potential role in the suppression of inflammatory response induced by *A. hydrophila* toxins through downmodulation of ROS production and CD14 and adhesion molecules expression, as well as increase of CD4^+^/CD8^+^ cell ratio.

## 1. Introduction

*Aeromonas* species are facultative aerobes and motile and gram negative bacteria. They are widely distributed in nature and involved in sepsis, wound infections, and food-borne gastroenteritis [1]. The virulence of* Aeromonas* (*A.*)* hydrophila* is based upon its extracellular proteins (ECP), such as aerolysins, hemolysins, enterotoxins, and proteolytic enzymes, as well as its extracellular polysaccharides (EPS) and lipopolysaccharides (LPS, endotoxin). Nam and Kiseong [2] showed that* Aeromonas* aerolysin can form channels by heptamerization of the host cell membrane. The pore channels impair epithelial integrity by promoting intestinal tight junction protein redistribution and thus affect wound closure [3, 4]. Meanwhile, the EPS of* Aeromonas* mediate the interaction between pathogenic bacteria and their environment through adhesion to the host cells [5, 6]. In particular,* A. hydrophila* infection rapidly alters a number of potentially critical lectins, chemokines, interleukins, and other mucosal factors in a manner predicted to enhance its ability to adhere to and invade host tissues [7]. An equally important nonfimbrial adhesion factor that has been implicated in the pathogenesis of* Aeromonas* spp. is LPS. As an adhesin, S-type LPS is indispensable for initial attachment of bacteria to host tissue and is necessary during infection events, where it protects bacteria from antimicrobial peptides and complement-mediated killing [8, 9].

CD14 is expressed on the surface of monocytes, macrophages, and neutrophils and occurs as a membrane-bound form and a soluble form [10, 11]. It has been implicated in the development and maturation of the innate immune system [12–15]. Several studies have reported the relationship between CD14 and its role in the polarisation of T lymphocytes into Th1 and Th2 subsets [16–20].

Classical immunoregulatory tissues control and determine the success of critical early steps in pathogenesis including microbe adhesion, entry, and replication [7]. Even when mucosal tissues are healthy, they are bathed in low levels of E-selectin, intercellular adhesion molecule 1 (ICAM-1), and interleukin 8 (IL-8). Of these, IL-8 forms a gradient of expression that is greatest near the bacteria/epithelial cell interface [21, 22]. E-selectin, meanwhile, is a membrane glycoprotein and is expressed by endothelial cells in order to mediate the adhesion of leukocytes. It is upregulated rapidly during inflammation, resulting in increased leukocyte-endothelial cell adhesion [23]. Adhesion molecules play important roles in cellular interactions during inflammatory responses. Expression of ICAM-1, for example, plays an important role in the adhesion of monocytes to endothelial cells [24].

Regarding flavonoids, these have metal chelating, free radical scavenging properties such as neutralization of the singlet oxygen and superoxide and inhibition of the hydrogen peroxide-induced lipid peroxidation (LPO) [25, 26]. Flavonoids inhibit the expression of isoforms of cyclooxygenase, inducible nitric oxide synthase, and lipooxygenase, which are responsible for the production of NO, prostanoids, and leukotrienes, as well as inflammatory mediators such as cytokines, chemokines, or adhesion molecules [27].

Hesperidin (HES) is a flavanone glycoside commonly found in the diet in citrus fruits or citrus fruit derived products [26, 28]. The anti-inflammatory effects of HES have been characterized* in vitro* in both rodent and human cell lines [29, 30]. The scavenging effect of free radicals associated with HES has been evidenced by different neurochemical and neurobehavioral parameters, with HES treatment appearing to reduce expression of proinflammatory mediators like inducible nitric oxide synthase (iNOS), TNF-*α*, and IL-1*β* [31, 32]. Recently, HES has been shown to exhibit pronounced immunological activities, serving to inhibit inflammatory cell infiltration and mucus hypersecretion in a murine model of asthma [33]. In addition, HES counteracted the upregulation of proinflammatory cytokines, such as the expression of TNF-*α* and IL-1*β*, in cerebral ischemia [31, 34, 35], as well as IL-8, IL-6, IL-12, and vascular cell adhesion molecule 1 (VCAM-1), in the case of acute lung inflammation induced by LPS* in vivo* [36].

The aim of the present study was to investigate the antiadhesion and anti-inflammatory role of HES in the case of gastrointestinal* Aeromonas* infection in a murine model.

## 2. Materials and Methods

### 2.1. Bacteria and Growth Conditions

A standard* A. hydrophila* strain (ATCC; catalogue number 7966) was kindly provided by the Fish Department, Faculty of Veterinary, Cairo University, Giza, Egypt. The bacterium was maintained and subcultured three times before the experiments. Briefly, 100 *μ*L of* A. hydrophila *was inoculated into 150 mL of a liquid peptone broth (Oxoid) and incubated for 30°C for 24 h with continuous shaking at 250 rpm. The harvested bacteria were centrifuged at 6000 g for 10 min and the dried pellet was suspended twice in phosphate-buffered saline (PBS) to the final dose of 2 × 10^8^ CFU/mL.

#### 2.1.1. Preparation of* A. hydrophila* Lipopolysaccharides (A-LPS)

LPS was prepared as described by Westphal and Jann [37]. Briefly, the bacteriawere inoculated in 250 mL of a Luria Bertani (LB) broth and incubated for 24 h at 30°C on a shaker at 250 rpm. The culture was then centrifuged at 10000 rpm for 10 min at 4°C, resuspended in 16.6 mL of TAE buffer (40 mM Tris-acetate, pH.8.5; 2 mM EDTA), and then mixed with 33.2 mL alkaline solution (containing 3 g of SDS, 0.6 g of Trizma (Sigma), and 160 mL of 2 N NaOH in 1000 mL of water). The suspension was heated at 55 to 60°C for 70 min and then mixed with phenol and chloroform in the ratio of 1 : 1 (V/V). The mixture was spun at 10 000 rpm for 10 min at 4°C and the supernatant obtained was mixed with 33.2 mL of water and 8.3 mL of 3 M sodium acetate buffer (pH 5.2). LPS was precipitated by adding twice the volume of ethanol. The precipitate was dissolved in 33.2 mL of 50 mM Tris-HCl, pH 8.0 (Sigma), and 100 mM sodium acetate, mixed well, and was then reprecipitated with twice the volume of ethanol. The combined water extract was dialyzed for 2–4 days against distilled water and then freeze-dried.

#### 2.1.2. Preparation of* A. hydrophila* Extracellular Proteins (A-ECP)

The bacterial isolate was grown overnight in 5 mL LB broth for preculturing. 100 *μ*L of this culture suspension (inoculum) was added to 50 mL LB broth and incubated overnight at 37°C at a shaker speed of 200 rpm. The culture suspension was harvested at 5000 rpm at 4°C for 15 min. The supernatant was precipitated by the addition of 10% (w/v) trichloroacetic acid with overnight incubation at 4°C. Further centrifugation at 11000 rpm for 20 minutes resulted in a pellet containing extracellular proteins which was suspended in 50 *μ*L of 1 M Tris-HCl buffer (pH 8) and dialyzed overnight against the same buffer. The freeze-dried protein content was determined as described by Lowry et al. [38]. The purified protein was ascertained as endotoxin-free with the limulus amebocyte lysate (LAL) test.

### 2.2. Animals

Male MF1 albino mice (7-8 weeks old; weighing 20–25 g; King Fahd Specialist Medical Centre, Jeddah, KSA) were used in the experiments and housed in a barrier room under standard conditions. The animals were kept in wire-mesh polycarbonate cages with autoclaved bedding, were acclimatized to laboratory conditions (12 h dark: 12 h light cycles; 24.0 ± 1.0°C), and had free access to food and water ad libitum. The food containers were refilled daily with fresh standard diet and were fitted with bars to reduce losses. Routine clinical observations and body weight were measured regularly throughout the experiments. Animal use and the care protocol were approved by the Research Ethics Committee, College of Medicine, Taif University, Saudi Arabia.

### 2.3. Natural Products

Hesperidin (HES) used in this study was of analytical grade and purchased from Sigma Chemical Co. (St. Louis, Missouri, USA) and dissolved in 1% dimethyl sulphoxide (DMSO) immediately before use.

### 2.4. Experiment Design

For* in vivo* studies, mice were randomly assigned to four groups (*n* = 10/group) as follows. (1) Control group (C) received only the standard diet, had free access to sterile water, and was orally fed with PBS (pH 7.4; 0.2 mL/mice) using intragastric intubation at intervals parallel to the treated groups. (2) Bacteria group (B) was orally fed once per week with bacterial suspension of* A. hydrophila* (0.2 mL containing 2 × 10^8^ CFU/mouse) for four consecutive weeks. This dose was selected according to Abuelsaad et al. [39]. (3) In infected-treated group (B-HES), bacteria-infected mice were orally fed with 250 mg HES/kg/week for four consecutive weeks according to Abuelsaad et al. [39]. At the end of week four following infection and treatment, blood was collected from the retroorbital sinus into sodium citrate (0.38%).

### 2.5. Quantification of Phagocytic Index in Blood

Phagocytic ability of neutrophils was performed according to a modified version of a previously described assay for the intracellular conversion of nitroblue tetrazolium (NBT) to formazan by superoxide anion [40, 41]. Briefly, 0.1 mL of blood was mixed with 0.1 mL of 0.2% NBT solution (Sigma) in sterile plastic test tubes for 30 min at room temperature. The formazan content of the cells was then solubilized with 960 *μ*L 2 M KOH and 1120 *μ*L DMSO, and the extinction was measured spectrophotometrically in 1 cm cuvettes at optical density (OD) of the cells was 630 nm. Values of the extinction were transposed according to a standard curve into mg NBT formate per 1 mL of blood. A standard curve was prepared by adding KOH and DMSO to known amounts of NBT. As a positive control, 100 mM hydrogen peroxide was added to cells and the amount of formazan formed was measured. At the same time, the total number of the leukocytes was examined in order to calculate the absolute number of blood neutrophils. Individual mouse blood samples were applied in triplicate, and the mean was calculated. The NBT index was determined by using the following equation:

(1) Phagocytic  index  in  blood  (NBT  conversion) =mg  of  NBT  formate/1 mL  bloodNeutrophil  count  in  thousands.

### 2.6. Quantification of Reactive Oxygen Species in Intestinal Tissues

Intracellular conversion of NBT to formazan by superoxide anion (O_2_^•−^) was used to measure the generation of reactive oxygen species [40–42]. About 0.1 mL of intestinal tissue homogenate was incubated with 0.1 mL of 10 *μ*M NBT (Sigma) for 30 min to allow O_2_^•^ generated from the collected intestinal tissues to reduce NBT to formazan. The formazan content of was then solubilized with 960 *μ*L 2 M KOH and 1120 *μ*L DMSO determined spectrophotometrically at 630 nm against a mixture of KOH and DMSO as a blank. As a positive control, 100 *μ*M H_2_O_2_ was added to cells and the amount of formazan formed was measured. Standard curves of NBT (0–10 *μ*M) were constructed by using the mixture as a vehicle. The SOD-inhibitable NBT reduction was calculated by subtracting the average of the negative controls from all other samples. Final O_2_^•^ production was expressed as nmoles of NBT per milligram protein per 30 min incubation time. Individual mouse samples were applied in triplicate and the mean was calculated.

### 2.7. Flow Cytometry (FACS) Analysis for CD Markers

#### 2.7.1. Total Lymphocytes and Monocytes Isolation

Small slices from intestine tissues were homogenized using 40 *μ*m cell strainers (BD Falcon, Bedford, MA). Red blood cells were osmotically lysed using lysis buffer containing 0.165 M NH_4_Cl_2_. Lymphocytes are resolved from other white blood cells (granulocytes, monocytes) based on density gradient centrifugation using lymphocyte separation medium (LSM 1077; PAA Laboratories, Germany) as described by Badr et al. [43]. Monocytes were isolated from lymphocytes to evaluate CD14 expression by positive selection using magnetic CD14 microbeads (human; Cat number 130-050-201, Miltenyi Biotec, Germany) as described by Neu et al. [44].

Lymphocytes and monocytes were washed with phosphate-buffered saline (PBS, pH 7.4), counted using trypan blue exclusion test, and cultured in complete R-10 medium (RPMI 1640 medium supplemented with 10% FCS, 2 mM L-glutamine, 100 IU/mL penicillin, 100 *μ*g/mL streptomycin, 1 mM sodium pyruvate, and 50 *μ*M 2-mercaptoethanol). The purity of cells was assessed using flow cytometry and was greater than 90%. Cells were cultured in R-10 medium.

#### 2.7.2. Antibodies and Flow Cytometry

Cells were stained with mAbs and analyzed using a FACSCalibur (BD, Franklin Lakes, NJ) according to Neu et al. [44]. Briefly, purified lymphocytes and monocytes from intestinal tissues (1 × 10^6^ cells/50 *μ*L PBS) were washed once with washing buffer (3% (v/v) FBS and 0.1% (w/v) NaN_3_ in PBS), resuspended in blocking buffer (3% (v/v) FBS; 5% (v/v) normal human AB serum, Cat number C11-020; PAA Laboratories, Germany; and 0.1% NaN_3_ (w/v) in PBS) with purified CD16/CD32 FccII/III mAb (AbD Serotec Co., USA) to prevent nonspecific binding. Subsequently, cells were incubated with mAb for 20 min at room temperature in dark area with the following Fluor-conjugated FITC rat anti-mouse antibodies purchased from AbD Serotec Co., USA, as follows: CD3-FITC, CD4-FITC (Cat numbers MCA500FT and MCA1767FT, resp.), and PE-conjugated anti-CD8 (CAT# MCA1768PE) and anti-CD14 (CAT# MCA2745PE). Subsequently, cells were washed, fixed in paraformaldehyde (PFA; 4% (v/v) in PBS; Sigma-Aldrich, Germany), and stored at 4°C in washing buffer until further use.

A FACS Calibur flow cytometer was used for data acquisition, with Diva software (BD Biosciences) for data analysis. After gating on viable cells, 10,000 events per sample were analyzed. For each marker, the threshold of positivity was defined beyond the nonspecific binding observed in the presence of a relevant control mAb.

### 2.8. Expression of Adhesion Molecules on HUVECs and RAW Macrophage

Human umbilical vein endothelial cells (HUVECs) and RAW macrophage cell lines were obtained and cultured as described by Takami et al. [45] and Leitinger et al. [46]. Monolayer of HUVECs and RAW cells (passages 4–6) was incubated with 100, 150, or 200 *μ*M/mL HES for two hours in the presence or absence of* Aeromonas* LPS (100 ng/mL) or* Aeromonas* ECP (100 ng/mL) in medium 199 (M199) containing 20% supplemented fetal bovine serum (FBS), 1 unit/mL heparin, 50 *μ*g/mL bovine endothelial cell growth supplement (Technoclone, Vienna, Austria), 2 mM glutamine, 100 units/mL penicillin, and 100 *μ*g/mL streptomycin. Antibodies for whole-cell ELISAs using cell-surface-expressed method for E-selectin or intercellular adhesion molecule 1 (ICAM-1) were obtained from R&D Systems (Minneapolis, Minnesota). Detection is performed using goat anti-mouse antibody conjugated to peroxidase. O-Phenylene diamine (OPD, Sigma) was used for colour development, the reaction was stopped using 3 M H_2_SO_4_, and optical density (OD) was read at 492 nm using a microtiter plate reader (ANTHOS, Salzburg, Austria).

### 2.9. Statistical Analysis

Analysis of variance on SPSS software package (version 16) was used to test the present data. One-way analysis of variance (ANOVA) was used to study the significant differences. In the case of significant difference, the multiple range comparisons (Duncan's test) was selected from the post hoc window on the same statistical package to detect the distinct variance between means. For further analysis, all values are given as the means ± SD. Differences with *P* < 0.05 were considered statistically significant.

## 3. Results

### 3.1. Changes in Body and Organ Weights

**Figure 1:**
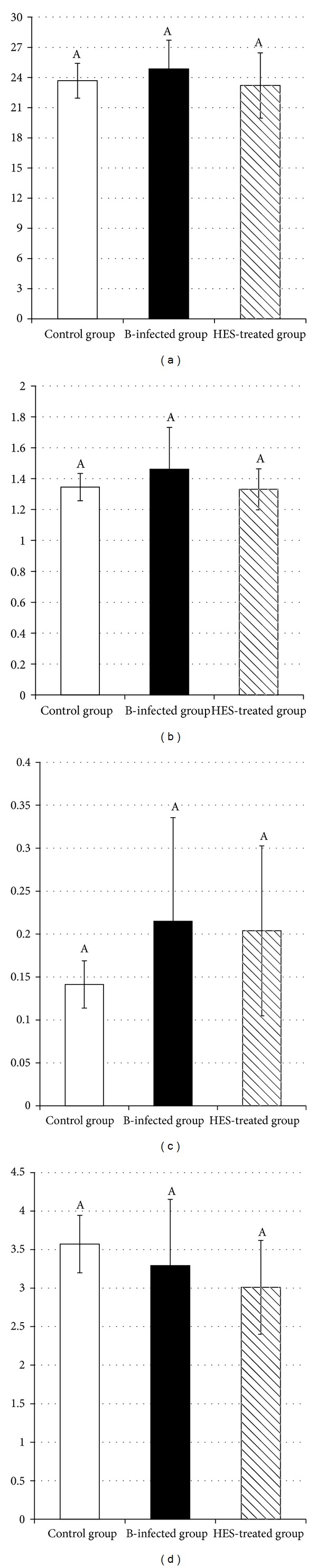
*In vivo* effect of hesperidin inoculation on body weight (a), liver weight/g (b), spleen weight/g (c), and intestine weight/g (d). Mice were infected, each with 2 × 10^8^ CFU of* Aeromonas hydrophila* per week for four consecutive weeks (B-infected group), and treated simultaneously with hesperidin at a dose of 250 mg/kg/week for four consecutive weeks (HES-treated group). At the end of week 4 following exposure and treatment, mice were sacrificed and weighted, the liver, spleen, and intestine were weighted, phagocytic activity was estimated in fresh blood, and intestinal ROS production was evaluated in intestinal homogenate. Values not sharing common superscripts denote significant differences.

Concerning changes in body and organ weights, Figure 1(a) shows that body weight did not significantly (*P* > 0.05) change between the groups (24.86 ± 2.847 g in the infected group versus 23.682 ± 1.728 and 23.211 ± 3.244 g in the control and HES-treated groups, resp.). Liver weight recorded a nonsignificant increase (*P* > 0.05, Figure 1(b)) in the infected group (1.461 ± 0.271 g in the infected group versus 1.346 ± 0.028 and 1.331 ± 0.133 g in the control and HES-treated groups, resp.). Similarly, spleen weight (Figure 1(c)) showed a nonsignificant increase (*P* > 0.05) in the infected group (0.141 ± 0.028, 0.215 ± 0.121, and 0.204 ± 0.099 g for control, infected, and HES-treated groups, resp.). Meanwhile, the intestine weight (Figure 1(d)) was not significantly (*P* > 0.05) altered in the different groups (3.572 ± 0.373, 3.291 ± 0.861, and 3.010 ± 0.609 g for control, infected, and HES-treated groups, resp.).

### 3.2. Quantification of Phagocytic Activity and ROS Production

**Figure 2:**
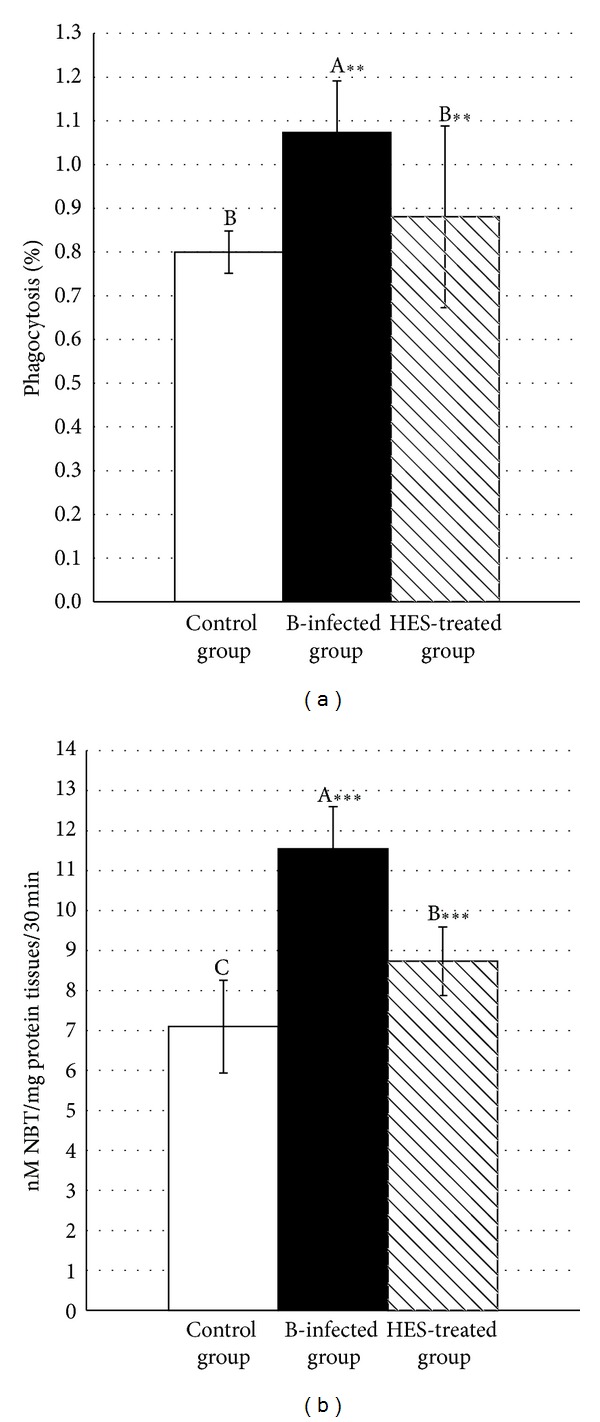
*In vivo* effect of hesperidin inoculation on phagocytic activities in blood (NBT index) (a) and intestinal reactive oxygen species (ROS) production (b). Mice were infected, each with 2 × 10^8^ CFU of* Aeromonas hydrophila* per week for four consecutive weeks (B-infected group), and treated simultaneously with hesperidin at a dose of 250 mg/kg/week for four consecutive weeks (HES-treated group). At the end of week 4 following exposure and treatment, mice were sacrificed and weighted, the liver, spleen, and intestine were weighted, phagocytic activity was estimated in fresh blood, and intestinal ROS production was evaluated in intestinal homogenate. Values not sharing common superscripts denote significant differences.

Regarding the quantification of the phagocytic ability of neutrophils in blood, Figure 2(a) shows that there was a highly significant (*P* < 0.001) increase in the* A. hydrophila*-infected group (1.073 ± 0.117%) in comparison to the control (0.80 ± 0.048%) and HES-treated (0.881 ± 0.208%) groups. This data should be discussed in parallel with the quantification of reactive oxygen species in intestinal tissues, as measured by the intracellular conversion of NBT to formazan by the superoxide anion (O_2_^•−^). Intestinal ROS production (nM NBT/mg protein tissues/30 min; Figure 2(b)) showed a highly significant (*P* < 0.001) increase in the infected group (11.545 ± 1.052 nM NBT/mg protein tissues/30 min) in comparison to the control (7.099 ± 1.161) and HES-treated (8.736 ± 0.86) groups.

### 3.3. Flow Cytometry (FACS) Analysis for CD Markers

**Figure 3:**
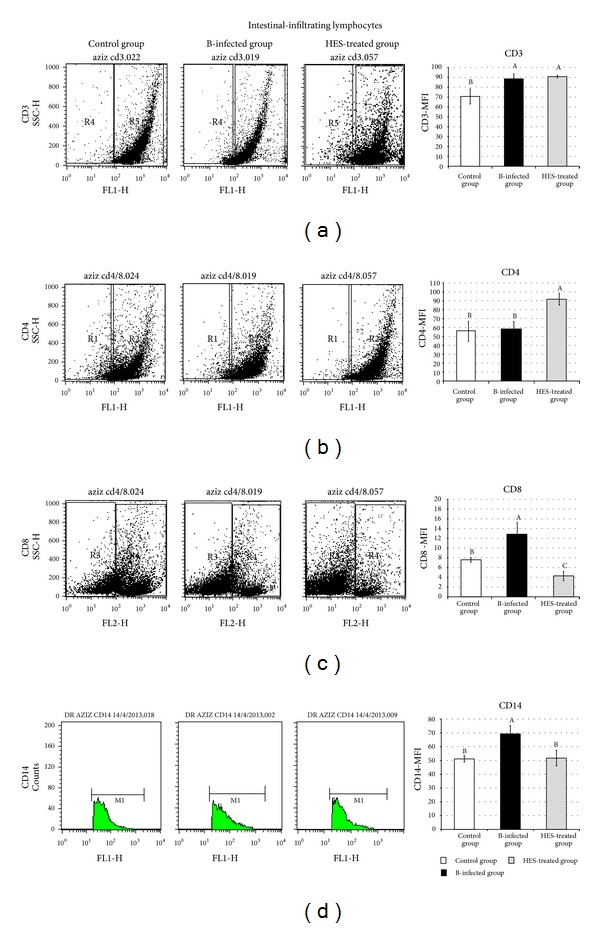
Representative dot plots of FACS analysis showing changes in Mean Fluorescence Index (MFI) of CD3^+^, CD4^+^, and CD8^+^ lymphocytes and CD14^+^ monocytes in intestinal infiltrating cells in different groups control group (C); bacteria-infected group (B); and bacteria treated with hesperidin group (HES-treated group). Data reported as Mean Fluorescence Index (MFI) ± standard deviation (SD). Values of the same parameter not sharing common superscripts denote significant differences.

Quantification of the CD markers of the intestinal infiltrating lymphocytes and monocytes obtained from a selection of mice is illustrated in Figure 3. The results showed that HES treatment significantly increased CD4^+^ T cells in the intestinal infiltrating lymphocytes (91.73 ± 6.55 with *P* < 0.001) versus 55.55 ± 11.10 and 58.45 ± 8.21 for the control and infected groups, respectively (Figure 3(b)). On the other hand,* A. hydrophila* infection induced a highly significant elevation in CD8^+^ T cells (*P* < 0.001), while HES treatment significantly suppressed this increase in the number of CD8^+^ T cells (7.600 ± 0.50; 12.858 ± 2.3; 4.290 ± 0.94 for control, bacteria-infected, and HES-treated groups, resp.) (Figure 3(c)). Taken together, the present data shows that the ratio of CD4^+^/CD8^+^ T lymphocytes in* A. hydrophila*-infected mice was significantly increased by HES treatment.

Moreover,* A. hydrophila* infection induced a highly significant expression of CD14^+^ on the surface on intestinal infiltrating monocytes (69.322 ± 5.91 with *P* < 0.001), while HES treatment significantly suppressed this elevation (51.168 ± 2.25 and 51.734 ± 5.67 for control and HES-treated groups, resp.) (Figure 3(d)).

### 3.4. Expression of Adhesion Molecules Using Modified Cell ELISA

Expression of E-selectin was explored* in vitro* by modified cell enzyme linked immunosorbent assay (ELISA). Pretreatment of human umbilical vein endothelial cells (HUVECs) with A-LPS (100 ng/mL) significantly increased the expression of both E-selectin and ICAM-1 (0.525 ± 0.082 and 1.519 ± 0.092, resp.), as shown in Table 1. Treatment of HUVECs with different concentrations of HES, meanwhile, significantly suppressed the A-LPS-induced expression of E-selectin (0.214 ± 0.007, 0.122 ± 0.002, and 0.225 0.031 for 100, 150, and 200 *μ*M HES/mL, resp.) and the expression of ICAM-1 (0.209 ± 0.011, 0.181 ± 0.016, and 0.145 ± 0.01 for 100, 150, and 200 *μ*M HES/mL, resp.), as shown in Table 1.

Moreover, A-ECP induced a highly significant expression (*P* < 0.001) of E-selectin and ICAM-1 (0.833 ± 0.068 and 1.491 ± 0.099, resp.), as shown in Table 1. HES treatment, on the other hand, significantly suppressed this A-ECP-induced expression of E-selectin (0.195 ± 0.052, 0.114 ± 0.002, and 0.136 ± 0.018 for 100, 150, and 200 *μ*M HES/mL, resp.) and that of ICAM-1 (0.143 ± 0.005,0.126 ± 0.01, and 0.096 ± 0.005 for 100, 150, and 200 *μ*M HES/mL, resp.) (Table 1).

The expression of ICAM-1 on RAW macrophage was explored* in vitro*, with the results set out in Table 1. Pretreatment of RAW cells with A-LPS and A-ECP (100 ng/mL) significantly increased the expression of ICAM-1 (1.452 ± 0.074 and 1.401 ± 0.063, resp.). The data showed that HES suppressed A-LPS-induced expression of ICAM-1 on RAW cells (1.224 ± 0.12, 1.096 ± 0.087, and 1.04 ± 0.212 for 100, 150, and 200 *μ*M HES/mL, resp.). Similarly, HES suppressed A-ECP-induced expression of ICAM-1 on RAW cells (1.148 ± 0.159, 1.061 ± 0.045, and 1.215 ± 0.029 for 100, 150, and 200 *μ*M HES/mL, resp.) (Table 1).

## 4. Discussion

Previously, it was reported that all mice injected i.p. with* Aeromonas hydrophila* had died within twenty days of infection [39]. Pretreatment with HES (250 mg/kg b.wt), however, was effective and significantly (*P* < 0.05) prolonged the survival of the mice beyond twenty days from infection [39]. Regarding body weight, the current study shows no significant difference in body, liver, spleen, and intestine weights. These findings are in accordance also with our previous* in vivo *study [39], which also showed no significant changes (*P* > 0.05) in body or intestine weights between the experimental groups.

The recorded nonsignificant elevation in spleen weight of both infected and HES-treated groups may be due to* Aeromonas* LPS which caused the releasing of secretory products from the activated circulating leukocytes and vascular endothelial cells, for example, TNF-*α* and free radicals. TNF-*α* activates a variety of tissue cells to release interleukin 8 (1L-8). 1L-8 enhanced the adhesion of leukocytes to endothelium and induced leukocytic degranulation and oxygen radical release, which causes endothelial cell necrosis [47]. Also, released free radicals may react around the blood vessels of the liver and develop hepatic injury by forming another radical peroxynitrite [48].

On activation by different antigens, the phagocytic cells from infected animals produced significantly higher ROS than those from noninfected animals, indicating the involvement of immune T cells. Previous data has shown that the bacterial LPS caused an increase in reactive nitrogen intermediates (RNI), reactive oxygen species (ROS), and their phagocytic index production. Excessive ROS could directly lead to cell damage and tissue injury by targeting various biomacromolecules, such as proteins, lipids, and DNA [49, 50]. The higher phagocytic activity shown here may be due to LPS-induced degranulation in macrophages, but, like allergens, it also stimulates the* de novo* synthesis and release of cytokines in these cells. Several* Aeromonas* infections are known to stimulate the robust host production of nitrite oxide radicals (NO) and ROS, leading to the loss of mitochondrial membrane potential and apoptosis [51].

Other reasons for the elevation in the phagocytic index and ROS production recorded in the present study may be due to aerolysin or cytotoxic enterotoxin (Act) secretions from* A. hydrophila* infection or the release of extracellular proteins. Aerolysin binds to cell surface structures and oligomerizes, forming channels that result in cell lysis [52]. Act is the most potent virulence factor in* A. hydrophila* strains, serving to bind and stimulate infiltration of phagocytic cells, for example, monocytes and macrophages, and induce the release of ROS [53, 54]. Recently, Act has been shown to recruit neutrophils in inflammatory diseases [55–58] and to upregulate macrophage inflammatory proteins* in vitro* [59]. On the other hand, the data from the present study clearly shows that HES treatment significantly reduced the elevation in ROS production that had been provoked by* A. hydrophila* infection. The antioxidant efficacy of HES may be attributed to its ability to inhibit ROS generation, including hydroxyl radical [60] and scavenging peroxynitrite radicals [61].

The significant increase in CD14 bearing cells as a result of* A. hydrophila* infection may be due to the release of LPS which may in turn induce responses by interacting with a soluble binding protein in serum that then binds with CD14 [62]. Also, LPS activate macrophages through CD14 [63]. CD14 is a multifunctional high-affinity pattern recognition receptor for bacterial endotoxins, LPS, and other bacterial wall components [20, 64]. CD14 binding of LPS is associated with a strong IL-12 response by antigen presenting cells [65, 66] and IL-12 is regarded as an obligatory signal for the maturation of naive T cells into Th1 cells [65]. Proliferation of mucosal lymphocytes, natural killer cells, and macrophages is stimulated by IL-12 [67, 68]. Li et al. [69, 70] predicted that potent downregulation of IL-2R*β* may be a key immunosuppressive strategy of* A. hydrophila* to facilitate successful infection of the skin mucosal surface. Recently, it was reported that subjects with allergic asthma have increased expression of CD14 after LPS inhalation [71]. The current study demonstrates that HES treatment downregulates CD14 expression on infiltrated cells in the intestinal tissues of* A. hydrophila*-infected mice and this may then reduce the inflammatory response caused by infection in such tissues.

The CD4^+^/CD8^+^ ratio is a reflection of immune system health. FACS assay showed that* A. hydrophila* infection dramatically decreased the percentage of CD4^+^/CD8^+^ cells in intestinal tissues. On the other hand, the CD4^+^/CD8^+^ ratio in the HES-treated group was significantly (*P* < 0.001) elevated after four weeks of treatment, indicating the progressive development of CD4^+^ cells. Previously, Lee et al. [72], in the context of a study on asthma, showed that effects of HES on lymphocyte subsets in lungs and bronchoalveolar lavage fluid (BALF) were characterised by an increase in the number of CD4^+^ helper T cells and a reduction in CD8^+^ cells.

The highly significant elevation in adhesion molecules observed after stimulation with* Aeromonas* LPS or A-ECP may be due to their direct effects in altering and disrupting the actin cytoskeleton of targeted cells so as to gain entry to and/or manipulate cellular immunity [2, 73]. These disruptions can themselves often lead to cell death at sites of infection [74]. In particular,* A. hydrophila* infection rapidly altered a number of potentially critical lectins, chemokines, interleukins, and other mucosal factors in a manner predicted to enhance its ability to adhere to and invade the host tissues [70]. Bacterial LPS and inflammatory cytokines, including TNF-*α*, IL-1, and IFN-*γ* stimulate ICAM-1 and VCAM mRNA accumulation and cell surface expression, although this mechanism is thought to promote tissue inflammation [75]. The upregulation of the gene expression of adhesion molecules in microvascular endothelial cells is an important step for the migration and accumulation of leukocytes at the site of inflammation, which play a critical role in organ damage during sepsis [23, 76]. Our data shows that HES downmodulates expression of E-selectin and ICAM-1 on both HUVECs and RAW macrophage. These results are in agreement with the findings of Nizamutdinova et al. [24] who found that HES suppresses ICAM-1 and VCAM-1 expression in TNF-*α*-treated HUVECs. These effects were caused by the inhibition of PI3 K/Akt and PKC signaling pathways. HES has also been reported to reduce the expression of IL-8, TNF*α*, IL-1*β*, IL-6, IL-12, ICAM-1, and VCAM-1 in the case of acute lung inflammation induced by LPS* in vivo* [36]. Moreover, it has been shown that pretreatment with HES could suppress infection-induced endotoxic shock in mice and reduce bacterial numbers during infection [77]. Also, the recorded amerolative effects of HES may result from the influx of neutrophils into the inflamed area, phagocytizing the bacteria and digesting them. This serves to activate different host defence mechanisms to both reduce bacterial numbers and counteract endotoxic shock [39].

The effect of HES on the expression of E-selectin and ICAM-1 is dose dependent, since 150 *μ*M of HES downregulated expressions of both E-selectin and ICAM-1 in comparison with 100 and 200 *μ*M HES. The molecular mechanisms by which HES attenuates expression of E-selectin and ICAM-1 are unclear and need further investigation. Previous studies have, however, suggested that several flavonoids, including HES, interact selectively with the mitogen-activated protein (MAP) kinase signalling pathway. The extracellular signal-regulated kinase (ERK) phosphorylation was involved in TNF-*α*-induced ICAM-1 expression and PI3 K/Akt and protein kinase C (PKC) was involved in TNF-*α*-induced VCAM-1 expression [24, 78]. HES can reduce TNF-*α*-induced VCAM-1 expression through the regulation of the Akt and PKC pathway; that is, it inhibits the adhesion of monocytes to endothelium [24]. In addition, the systemic administration of HES produced a marked reduction in the phosphorylation state of extracellular signal-regulated kinases 1/2 (ERK 1/2) in the cerebral cortex, cerebellum, and hippocampus [79].

In conclusion, the results of the present study indicate that HES, as one of natural flavonoids, effectively suppressed ROS production, the phagocytic index, expression of E-selectin and ICAM-1 induced by A-LPS and A-ECP stimulation. These findings predict that HES treatment may effectively suppress cytokine networking and alter the adherence of stimulated phagocytic cells to endothelial barrier cells during inflammation. In addition, the present study provides strong support for the anti-inflammatory activities of hesperidin.

## Figures and Tables

**Table 1:** *In vitro* effect of different concentrations of hesperidin on the expression of E-selectin and ICAM-1 on HUVECs and RAW cells in response to *Aeromonas hydrophila* antigen stimulation. Human umbilical vein endothelial cells (HUVECs) and RAW macrophage were incubated for 2 h with 100, 150, and 200 *μ*M/mL hesperidin in the presence or absence of *Aeromonas hydrophila* antigen (Ag), lipopolysaccharides (A-LPS, 100 ng/mL), and extracellular proteins (A-ECP, 100 ng/mL). Expression of E-selectin on HUVECs and intercellular adhesion molecule 1 (ICAM-1) on HUVECs and RAW macrophage were estimated by using modified cell ELISA. Data reported as mean optical density (OD) ± standard deviation (SD). Values of the same parameter not sharing common superscripts denote significant differences.

	A-LPS			A-ECP		
	E-selectin HUVECs	ICAM-1 HUVECs	ICAM-1 RAW	E-selectin HUVECs	ICAM-1 HUVECs	ICAM-1 RAW
Control	0.090 ± 0.007^d^	0.092 ± 0.003^d^	0.084 ± 0.002^e^	0.090 ± 0.007^d^	0.092 ± 0.003^d^	0.084 ± 0.002^e^
Ag	0.525 ± 0.082^a^	1.519 ± 0.092^a^	1.452 ± 0.074^a^	0.833 ± 0.068^a^	1.491 ± 0.099^a^	1.401 ± 0.063^a^
HES-100	0.114 ± 0.002^cd^	0.170 ± 0.053^c^	0.079 ± 0.009^e^	0.114 ± 0.002^d^	0.170 ± 0.053^ac^	0.079 ± 0.009^e^
HES-150	0.114 ± 0.001^cd^	0.795 ± 0.082^b^	0.273 ± 0.063^d^	0.114 ± 0.001^d^	0.795 ± 0.082^b^	0.273 ± 0.063^d^
HES-200	0.170 ± 0.028^bc^	0.145 ± 0.009^cd^	0.129 ± 0.047^e^	0.170 ± 0.028^bc^	0.145 ± 0.009^cd^	0.129 ± 0.047^e^
Ag + HES-100	0.214 ± 0.007^b^	0.209 ± 0.011^c^	1.224 ± 0.120^b^	0.195 ± 0.052^b^	0.143 ± 0.005^cd^	1.148 ± 0.159^bc^
Ag + HES-150	0.122 ± 0.002^d^	0.181 ± 0.016^c^	1.096 ± 0.087^bc^	0.114 ± 0.002^d^	0.126 ± 0.010^cd^	1.061 ± 0.045^c^
Ag + HES-200	0.225 ± 0.031^b^	0.145 ± 0.010^cd^	1.040 ± 0.212^c^	0.136 ± 0.018^cd^	0.096 ± 0.005^cd^	1.215 ± 0.029^bc^
*F* value	74.74	582.74	175.673	284.78	589.78	386.209
*P* value	0.0000	0.0000	0.0000	0.0000	0.0000	0.0000
